# Reactive oxygen species creation by laser-irradiated indocyanine green as photodynamic therapy modality: an in vitro study

**DOI:** 10.1007/s10103-023-03876-1

**Published:** 2023-09-14

**Authors:** Diaa Atta, Abdelrahman Elarif, Mohamed Al Bahrawy

**Affiliations:** 1 Spectroscopy Department, Physics Research Institute, National Research Centre, 33 El Behooth St., Dokki, Giza, 60014618 Egypt; 2 Nonlinear Optical Properties and Fluorescence Spectroscopy Unit, Physics Research Institute, National Research Centre, 33 El Behooth St., Dokki, Giza, 60014618 Egypt; 3 Department of Oral Medicine, Faculty of Dentistry, Future University, Fifth Settlement, New Cairo, Egypt; 4 Oral Medicine & Periodontology Department, Faculty of Dentistry, Ain Shams University, Abbassia, Cairo, Egypt

**Keywords:** Photodynamic therapy, Laser ablation, Photosensitizers, Indocyanine green, Laser spectroscopy

## Abstract

Applications of lasers in phototherapy have been the trend for the last few decades. The photodynamic therapy process normally depends on photosensitizers and laser beams. Through this study, indocyanine green has been used as a photosensitizer, which is normally activated using laser lines between 750 and 805 nm. The activity of the indocyanine green to do fluorescence by other pulsed laser sources has been tested by fluorescence technique, and it has been proven that the laser lines at 810, 940, and 980nm are able to excite the indocyanine green with different extents. The indocyanine green activation has been tested by several laser lines (810, 940, and 980 nm) commonly used as surgical lasers. The generated oxygen has been measured after irradiating the indocyanine green with the different laser lines. A comparison has been made between laser irradiation as a pinpoint and a broad beam. It is found that the wide beam is more effective in activating oxygen production. In the end, it is concluded that lines 810 and 940nm were effective in activating the used dye, while the 980nm activity did not show enough efficiency.

## Introduction

Photodynamic therapy (PDT) is a non-invasive approach to counteract adverse microorganisms [1, 2], including bacterial oral pathogens. In PDT, light sources could be utilized to activate a photosensitizing medium in the presence of oxygen to generate reactive oxygen species (ROS). ROS can quickly destroy any surrounding biomolecules [3, 4].

Photosensitizers (PS) are dyes composed of molecules capable of absorbing light energy and using it to promote chemical reactions in cells and the surrounding tissues. The ideal photosensitizer should have the following: (1) high degree of chemical purity; (2) photostability at normal room temperature; (3) photosensitive effect in the presence of a certain wavelength; (4) high photochemical activity; (5) the absorption bands should not overlap with the absorption bands of other objects in the body, including endogenous dyes such as melanin, hemoglobin, or oxyhemoglobin; (6) minimal cytotoxicity in the dark; (7) high solubility in the tissues of the body; (8) cost-effectiveness; and (9) simple synthesis and easy availability. One could summarize all the previous as follows: PS should be a good fluorophore valid for laser spectroscopy or laser ablation [5, 6].

Indocyanine green (ICG) is a fluorescent dye from the tricarbocyanine dye group. Its solubility in water makes it an appropriate dye to use inside the body. As previously mentioned in several studies, ICG has clear absorption peaks between 600 nm and 900 nm. Other absorption peaks in the UV region and blue region have been reported, but all are weak. It is frequently utilized as a contrast agent for imaging purposes, such as measuring plasma volume, cardiac output, ocular angiography, and capillary microscopy, and it is quite safe. ICG is a photosensitizer with maximum absorption and fluorescence at 800 nm wavelength [7].

Low-level laser, the photonic energy, and the PS have been postulated as valuable options for treating localized infectious diseases. PDT is based on the possibility of the selective destruction of pathological tissues with an accumulation of photosensitizer molecules. [8]

The in vitro bactericidal effect of a diode laser with photoablative energies, together with ICG, has been recently studied for the first time by Ahrari et al. (2020) [9], who have performed a simultaneous photoablative and photodynamic treatment. The presented data in that study suggested that simultaneous photoablative-photodynamic irradiation in the presence of ICG as a PS could be a promising therapy against a wide range of bacteria involved in periodontitis disease; this would shorten treatment time (concurrent photoablation and photodynamic vs. photoablation followed by multiple photodynamic cycles) without compromising pathogen efficacy [9].

After the accumulation of the PS inside the targeted tissue, the specific lesion is irradiated with a laser source of the proper wavelength. PDT is a viable option for other cancer treatments because of its ease of use, impressive clinic results, low cost, and lack of side effects [10].

Sources generating light in the red region of the spectrum (600–700 nm) are limited in FDA-approved PDT applications. In comparison to the red section of the spectrum, human tissue is relatively transparent to near-infrared light, which can reach deep tissues. As a result, one of the newest areas of research in photodynamic treatment is finding photosensitive compounds that function with near-infrared light [7].

The methods of analysis are very miscellaneous; the used techniques in PDT or in general in biological molecules vary from FTIR and Raman to molecular modeling and molecular dynamics [11–22]. Besides the fluorescence techniques, one of the most common methods of analysis is the measurement of total dissolved oxygen (TDO) by the polarographic sensors [23–25].

Most of the photodynamic activity of ICG conforms to a photothermal dissociation to release smaller molecules, which are locally cytotoxic per se. This is different from a type I reaction where oxygen-based radicals (ROS) are produced. Studies have shown that ICG activated by a NIR diode laser affects the load and biofilm formation ability of *Porphyromonas gingivalis*, as well as increasing the disinfection of the root canal system. Excited photosensitizer particles do not damage organic cell structures and react only with oxygen molecules dissolved in the cytoplasm. The novelty of this work is based on two axes. The first and most important is the availability of creating the oxygen radical from the dissolved ICG dye by new laser lines, which could be available as surgical tools. The second is the method of analysis, especially the usage of conductive electrodes to determine the total dissolved oxygen without the need of very complicated and expensive tools of analysis or any expensive detectors with ultra-high quantum efficiency in the infrared region, especially at the 1270-nm region (the emission band of the singlet oxygen). The aim of this study is to examine the effect of diode surgical laser wavelengths on activating ICG dye to liberate oxygen radicals as a proof of concept for a new PDT modality.

## Materials and methods

### Study design

An experimental parallel in vitro study where oxygen tension was measured in samples of ICG dyes purchased from Aurolab, India. The purchased ampule contained 25 mg of ICG powder dissolved in 5 ml of Milli-Q water. The prepared ICG solution was divided into three equal groups, and every group was exposed to a laser source at one of the three different wavelengths. The experiment was repeated in triplicate, and graphs were plotted according to the oxygen tension level after laser activation. The collected data was analyzed and compared statistically to determine the difference between the three groups.

### Laser setup

The phototherapy has been done using three different laser sources: a 940 nm pulsed laser from BIOLASE, USA, with a pulse duration of 10μs, a total power of 7 W, and a spot size of 0.2 mm. also 810 nm pulsed laser from Elexxion, Germany, with a pulse duration of 16μs, and the total power has been adjusted to 7 W. The last one is Denlase, China 980nm diode laser with a pulse duration of 5 ms, the total power has been adjusted to 7 W, and a spot size of 0.2 mm at the fiber end. In the case of the source, the power was larger than 7 W, either it was adjusted from the source to 7 W or an attenuator was utilized to minimize the output power down to 7 W.

Laser power has been adjusted using the BeamMaster BM3 beam profiler from Coherent, USA.

Samples have been exposed to the laser beam as pin spots, and a self-made beam expander has been utilized to broaden the laser beam to distribute the energy over a large area.

### Total dissolved oxygen (TDO)

The total dissolved oxygen has been measured using one of the simplest techniques, which is the polarographic sensor, which is commonly utilized in measuring TDO amounts. TDO molecules could be reduced on the surface of any noble metal substrate, like gold, platinum, or silver. Hence, the working substrate could act as a cathodic electrode.

In the presence of such an anodic electrode, electron flow takes place from the anode to the cathode. That electron flow is proportional and expresses the amount of oxygen molecules that were dissolved inside the solvent.

Once the electron flow is the driving factor in the mentioned process, such an applied voltage should start the process. That applied voltage has to have at least the magnitude of the standard redox potential (+401 mV) of the reaction at the cathodic electrode, with reversed polarity. This negative voltage, the polarization voltage, must be an absolute constant and has to be stabilized against a reference electrode.

The TDO has been measured using Orion Versa Star Pro pH/ISE Benchtop Multiparameter Meter from Thermo Scientific, USA.

### Absorption spectroscopy

The absorption spectra have been measured using UV/Vis/NIR spectrophotometer model V770 from Jasco Corporation, Tokyo, Japan. The samples have been diluted to 5μg/ml.

### Fluorescence experiment

Emission spectra have been recorded using a time-resolved spectrofluorometer from Edinburgh Instruments Ltd., Kirkton Campus, UK. The spectrofluorometer laser has been removed and replaced by surgical laser props with wavelengths of 840, 940, and 980 nm. The laser props have been adjusted to hit the sample in the sample holder, and an internal attenuator has been utilized to reduce the laser power to 5 mW to do not affect the InGaAs detector.

The raw ICG has been diluted to 5μg/ml to avoid self-absorption.

### Spectral properties of the ICG

**Fig. 1:**
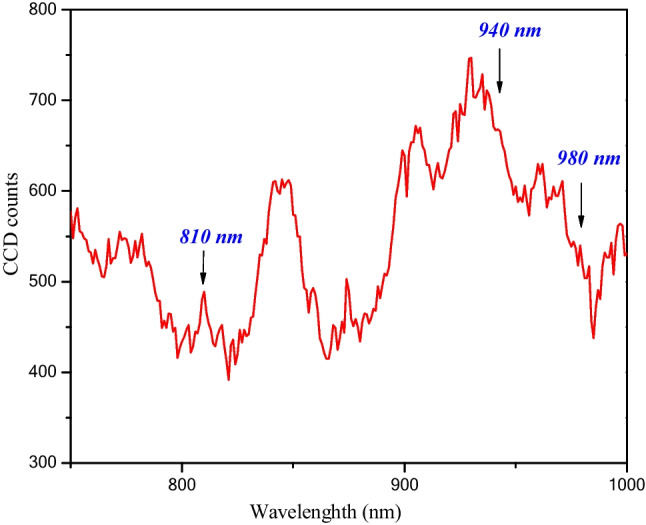
The excitation spectra of the used ICG powder

It is very important to test if the proposed lasers to initiate the reaction could excite the ICG molecules. In this regime, the excitation spectra between 750nm and 1000nm have been recorded to assure the suitability of the used laser heads. As it is clear in Fig. 1, the excitation spectra of the ICG confirm the suitability of the three laser heads used to excite the ICG as an initiator for the oxygen production.

## Results and discussion

Because free oxygen is the main core of PDT, which is the factor that primarily destroys microbes [26], it has been chosen to measure Oxygen tension in ICG solution as an indicator about the efficacy of laser energy to activate the dye.

As it is known, ICG has a high maximum absorption in the NIR region [27], which would be beneficial for PDT for many reasons. First, this region is suitable for soft tissue cutting, and so a single apparatus is used for both soft tissue laser surgeries and PDT. Second, the waves in the IR region have high penetrativity into the tissues [28].

**Fig. 2:**
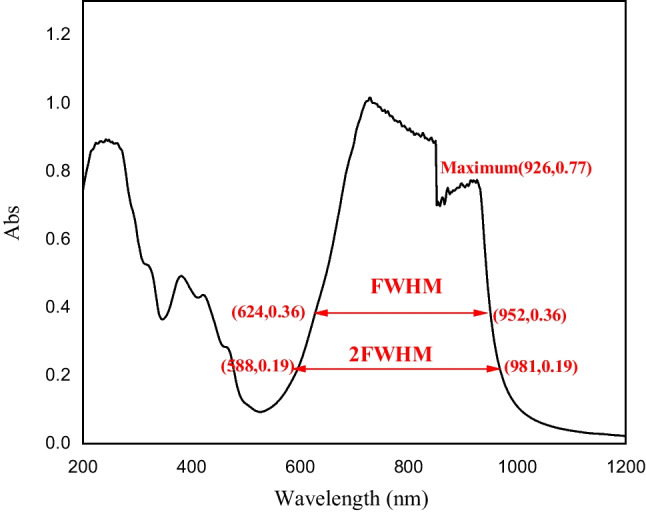
The absorption spectra of ICG as it is stated in the spectra the maximum coordinates was (926, 0.77), the full with at half maximum (FWHM) in between 624 nm and 952 nm, and the double FWHM was in between 588 nm and 981nm

First of all, it is famous that the ICG has an excitation band in between 750 and 800 nm, which emits at 830nm [28], so we should first study if the used wavelengths are able to excite the ICG or not. For this purpose, the absorption spectra have been measured as presented in Fig. 2. It is clear from the figure that the ICG has a wide excitation band with a FWHM between 624 nm and 950 nm. If we go down to 2 FWHM, the excitation region gets extended from 588nm to 981 nm, but with a very low absorbance of around 0.19.

**Fig. 3:**
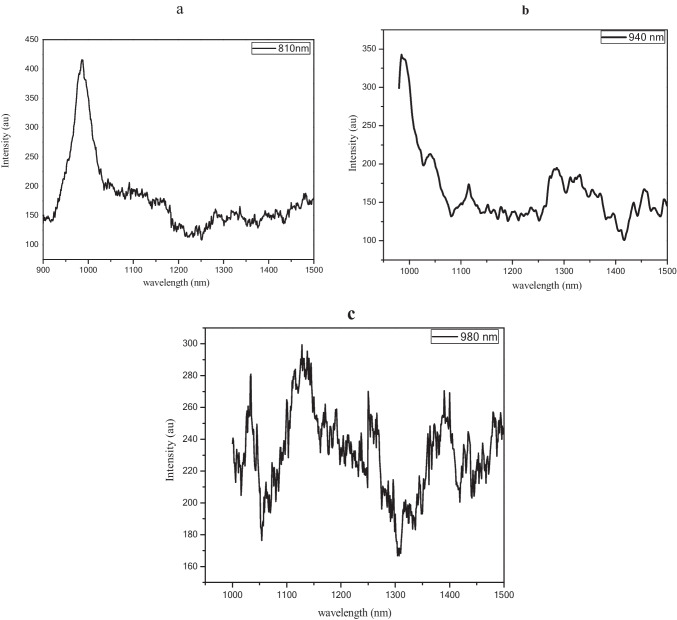
The emission spectra of ICG after excited by pulsed laser with wavelength **a** 840nm, **b** 940 nm, and **c** 980 nm

According to the absorption spectra, the laser-based photoluminescence spectra have been recorded depending on the mentioned surgery lasers as excitation sources. Figure 3 presents the emission spectra of ICG after being excited at 840nm, 940 nm, and 980 nm in sections a, b, and c of Fig. 3.

It is clear that both 840 and 940nm lasers could excite ICG approximately with the same amount of intensity, while the 980nm laser could hardly excite the ICG solution.

To confirm that the released oxygen is not resulting from irradiating the water molecules, the TDO has been measured for both Milli-Q water and a solution of 5 mg/ml ICG before and after the irradiation process at 810nm, 940nm, and 980 nm.

As it is presented in Table 1, it is clear that there is a fluctuation of the measured TDO values by changing the laser wavelength, but it did not exceed 0.08% and is in the range of instrumental error; hence, one could say that the used lasers did not free any oxygen.

**Table 1:** The average value and the standard deviation of the total dissolved oxygen before and after laser deposition with both pinpoint and expanded laser beam

Sample	Wavelength (nm)	TDO (μg/ml)							
		Expanded laser beam				Pinpoint laser			
		Before		After		Before		After	
		Ave	St. div.	Ave	St. div.	Ave	St. div.	Ave	St. div.
Pure water	810	1.32	0.059	1.45	0.043	1.38	0.008	1.35	0.055
	940	1.32	0.054	1.45	0.064	1.38	0.016	1.35	0.054
	980	1.3	0.078	1.35	0.057	1.31	0.145	1.29	0.076
Indocyanine green (ICG)	810	6	0.672	47	0.455	3.6	0.041	21	0.053
	940	17	0.927	30	0.589	8	0.07	13.56	0.571
	980	1.3	0.078	1.5	0.041	1.25	0.037	1.32	0.073

On the other hand, the ICG shows a dramatically increased level of dissolved oxygen after irradiation with 810nm and 940 nm lasers. While the 980nm one is not efficient at releasing oxygen like the others, this result is consistent with the absorption spectra and the emission of the ICG as presented in Figs. 2 and 3c.

Regarding the exposed area and the laser beam shaping, it is clear that the enlarged laser beam affected the oxygen generation more than the pinpoint source; this could be due to the increase in exposed molecules.

**Fig. 4:**
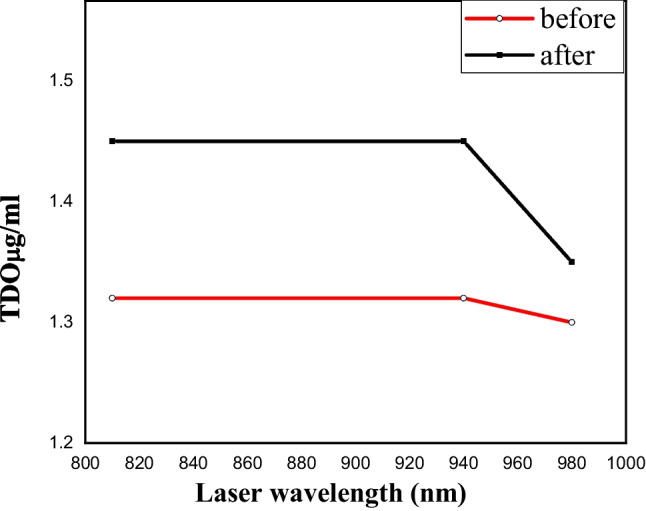
The amount of the TDO in the bi-distilled water before (red line) and after (black line) exposed to pinpoint different lasers with wavelengths 810, 940, and 980 nm

While there were no vast changes in the TDO values measured for Milli-Q water in both expanded and pinpoint laser, the pinpoint case has been presented in Fig. 4.

**Fig. 5:**
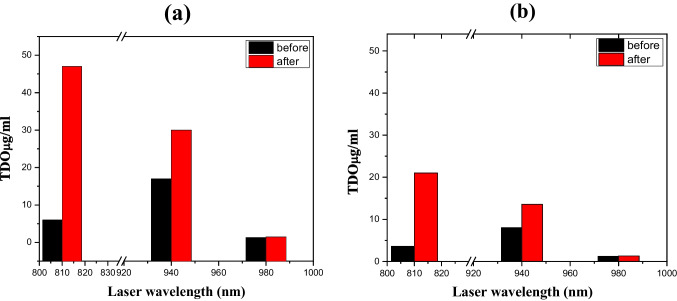
The amount of the TDO in the ICG before (black bar) and after (red bar) exposed to **a** broad and **b** pinpoint different lasers with wavelengths 810, 940, and 980 nm

As it is clear from Fig. 5a, b, one could easily notice that the measured TDO after irradiating with a broadened laser beam increases so much, especially if compared with that irradiated with a pinpoint laser.

This difference is normal because, by broadening the laser beam, the exposed area to the laser becomes larger and the rate of producing oxygen becomes greater. Several benefits could be gained from enlarging the cross-section area of the used laser beam. The first is that these lasers are surgical and designed to cut tissue; if someone uses them to activate ICG, it will harm the tissue, but broadening the beam reduces the laser power density. In addition, that size expansion reduces the scattering, which could easily take place as Raman signals [29, 30], which affects the oxygen production efficiency. The outlook of this work contains more intensive molecular modeling and electrostatic potential that could be helpful in understanding the reaction mechanism and providing the opportunity to enhance oxygen release [19, 31].

## Conclusion

It was expected that 810 nm would be effective in activating ICG dye and result in rapid liberation of free oxygen radicals as it is very near the normally used laser sources. It was skeptical about the ability of the 940-nm and 980-nm wavelengths to activate ICG and unleash oxygen free radicals because these wavelengths are away from ICG’s designated activation spectrum, but the findings were unexpected, as the 940nm laser was also able to activate the ICG dye and release oxygen. It has been proven that a 980-nm laser is not able to liberate oxygen with acceptable rate.

Within the scope of our research, an investigation of different diode laser wavelength effects on ICG and PS is necessary in order to prove more than the usual laser appliances that could be used for surgical ablation modalities and PDT at the same time. It is recommended to broaden the laser beam during the PDT process. From the current study, it is recommended to make more efforts to investigate ICG dye and its chemically modified structures in order to use it in even more treatment modalities, as it is expected for this dye to have untapped potential that could be used for antimicrobial and anticancer treatment.

The final output is the suitability of the 940-nm and 810-nm surgical lasers to activate the oxygen liberation from the ICG photoreaction. The necessity of enlarging the surgical beam diameter reduces the power density and distributes the laser energy to an extent that does not harm the tissues if it is required to be used during the activation of ICG.

## Data Availability

The datasets used and/or analyzed during the current study are available from the corresponding author on reasonable request.

## Abbreviations

PDT: Photodynamic therapy
ROS: Reactive oxygen species
PS: Photosensitizers
ICG: Indocyanine green
TDO: Total dissolved oxygen
